# Association of physical activity and air pollution exposure with the risk of type 2 diabetes: a large population-based prospective cohort study

**DOI:** 10.1186/s12940-022-00922-3

**Published:** 2022-11-06

**Authors:** Zhi-Hao Li, Wen-Fang Zhong, Xi-Ru Zhang, Vincent CH Chung, Wei-Qi Song, Qing Chen, Xiao-Meng Wang, Qing-Mei Huang, Dong Shen, Pei-Dong Zhang, Dan Liu, Yu-Jie Zhang, Pei-Liang Chen, Xin Cheng, Hai-Lian Yang, Miao-Chun Cai, Xiang Gao, Virginia Byers Kraus, Chen Mao

**Affiliations:** 1 Department of Epidemiology, School of Public Health, Southern Medical University, 510515 Guangzhou, Guangdong China; 2 Microbiome Medicine Center, Department of Laboratory Medicine, Zhujiang Hospital, Southern Medical University, Guangzhou, Guangdong China; 3 Jockey Club School of Public Health and Primary Care, The Chinese University of Hong Kong, Hong Kong, China; 4 Department of Nutritional Sciences, The Pennsylvania State University, University Park, PA University Park, USA; 5 Duke Molecular Physiology Institute, Division of Rheumatology, Department of Medicine, Duke University School of Medicine, Durham, NC USA

**Keywords:** Physical activity, Air pollution, Type 2 diabetes, Cohort study

## Abstract

**Background:**

The interplay between physical activity (PA) and air pollution in relation to type 2 diabetes (T2D) remains largely unknown. Based on a large population-based cohort study, this study aimed to examine whether the benefits of PA with respect to the risk of T2D are moderated by exposure to air pollution.

**Methods:**

UK Biobank participants (*n* = 359,153) without diabetes at baseline were included. Information on PA was obtained using the International Physical Activity Questionnaire short form. Exposure to air pollution, including PM_2.5_, PM_coarse_ (PM_2.5−10_), PM_10_, and NO_2,_ was estimated from land use regression models. Cox regression models were used to estimate the hazard ratios (HRs) and 95% confidence intervals (95% CIs).

**Results:**

During a median of 8.9 years of follow-up, 13,706 T2D events were recorded. Compared with a low PA level, the HRs for the risk of T2D among individuals with moderate and high PA were 0.82 (95% CI, 0.79–0.86) and 0.73 (95% CI, 0.70–0.77), respectively. Compared with low levels of air pollution, the HRs for risk of T2D for high levels of air pollution (PM_2.5_, PM_coarse_, PM_10_, and NO_2_) were 1.19 (1.14–1.24), 1.06 (1.02–1.11), 1.13 (1.08–1.18), and 1.19 (1.14–1.24), respectively. There was no effect modification of the associations between PA and T2D by air pollution (all *P*-interactions > 0.05). The inverse associations between PA and T2D in each air pollution stratum were generally consistent (all *P* for trend < 0.05).

**Conclusion:**

A higher PA and lower air pollution level were independently associated with a lower risk of T2D. The beneficial effects of PA on T2D generally remained stable among participants exposed to different levels of air pollution. Further studies are needed to replicate our findings in moderately and severely polluted areas.

**Supplementary Information:**

The online version contains supplementary material available at 10.1186/s12940-022-00922-3.

## Background

The global burden of type 2 diabetes (T2D) has increased over the past few decades, and its prevention is a public health priority [1]. Physical activity (PA) has been suggested to play an important role in the prevention of T2D and related morbidities [2–4]. In contrast, exposure to air pollution has been associated with an elevated risk of T2D [5–7]. Because PA increases the respiration rate, the intake of polluted air during outdoor PA may increase considerably and potentially intensify the detrimental health effects of air pollution. Therefore, it is of great public health interest to examine the tradeoffs between the health benefits of PA and the intensified harmful effects of air pollution during PA on T2D.

To date, only two studies have investigated the joint effect of PA and air pollution on diabetes [8, 9]. In a study of older Korean adults, Kim et al. [8] found that the benefits of PA on diabetes outweighed the risks related to ambient particulate matter (PM) exposure. This study examined only the weekly frequency of moderate to vigorous PA, which might not fully represent the amount of PA. Another study showed that habitual physical activity can reduce the risk of diabetes regardless of the levels of PM ≤ 2.5 μm (PM_2.5_) exposure [9]. To our knowledge, no study has examined the combined effects of PA and exposure to traffic-related air pollution, such as nitrogen dioxide (NO_2_), on the risk of T2D to date.

To address this knowledge gap, we used data from the UK Biobank, a large-scale prospective, population-based cohort study, to analyze associations of the frequency and duration of PA, long-term exposure to air pollution, and their joint effect with the risk of T2D. We assessed exposure to PM_2.5_, PM ≤ 10 μm (PM_10_), PM 2.5–10 μm (PM_coarse_), and NO_2_ to comprehensively examine the effects of air pollution on T2D. Furthermore, due to the suggestions of previous studies that genetic variations may modify associations between environmental factors and the risk of T2D [10, 11], we also examined interactions between PA or air pollution and genetic risk for T2D in this study.

## Methods

### Study design and participants

Between April 2007 and December 2010, the UK Biobank recruited 502,536 participants aged 40–69 years who attended one of 22 assessment centers across England, Wales, and Scotland [12]. Participants completed a touch screen questionnaire, had physical measurements taken, and provided biological samples, as described in detail elsewhere [13]. Ethical approval for the UK Biobank study was obtained from the North West Multi-centre Research Ethics Committee (06/MRE08/65), and all participants provided written informed consent.

We excluded 1299 participants who subsequently withdrew from the study, 132,920 participants who had incomplete information (99,907 with missing PA data and 33,013 with missing information for any of the air pollution exposures), and 9164 participants who had T2D at baseline. Our primary analyses included 359,153 participants. Participants (*n* = 7968) with missing genetic data were excluded from the genetic analyses. A flowchart of the study sample selection process is presented in Supplementary Fig. 1. There were no significant differences in baseline characteristics between the included individuals and the total population of the UK Biobank (Supplementary Table 1S).

### Assessment of physical activity

PA assessment was based on self-reports at baseline and obtained using the International Physical Activity Questionnaire (IPAQ) short form, which includes 6 questions about the frequencies and durations of three types of activities (walking, moderate-intensity activities, and vigorous activities) performed in a typical week [14]. Data were analyzed in accordance with the IPAQ scoring protocol. PA was computed in metabolic equivalent of task minutes per week (MET-min/wk), which could effectively measure the overall PA level of the participants and was then categorized as low (< 600 MET-min/wk), moderate (600–3000 MET-min/wk), or high (> 3000 MET-min/wk) PA [15]. Both categorical and continuous PA variables were used for the data analyses.

### Assessment of air pollution

The annual average concentrations of PM_2.5_, PM_coarse_, PM_10_, and NO_2_ were calculated using a land use regression (LUR) model developed by the European Study of Cohorts and Air Pollution Effects (ESCAPE) [16] and linked to the geocoded residential addresses of UK Biobank participants. The LUR model calculated the spatial variations in the annual average air pollutant concentrations at the participants’ home addresses, which were provided at the baseline visit, using predictor variables obtained from a geographic information system such as traffic, land use, and topography. Because of the use of a high-resolution European map [17], annual concentration data for PM_10_ and NO_2_ were available for several years (2007 and 2010 for PM_10_ and 2005, 2006, 2007, and 2010 for NO_2_) in the UK Biobank, therefore, we averaged the obtained values to obtain the air pollutant concentrations of PM_10_ and NO_2_. All other particulate matter (PM_2.5_ and PM_coarse_) exposure data were available for a single year only (2010) in the UK Biobank. Participants were divided into 3 categories on the basis of tertile cutoffs for each air pollution concentration: low PM_2.5_ (< 9.5 µg/m^3^), moderate PM_2.5_ (9.5–10.3 µg/m^3^), and high PM_2.5_ (> 10.3 µg/m^3^); low PM_coarse_ (< 5.9 µg/m^3^), moderate PM_coarse_ (5.9–6.4 µg/m^3^), and high PM_coarse_ (> 6.4 µg/m^3^); low PM_10_ (< 18.4 µg/m^3^), moderate PM_10_ (18.4–19.9 µg/m^3^), and high PM_10_ (> 19.9 µg/m^3^); and low NO_2_ (< 24.6 µg/m^3^), moderate NO_2_ (24.6–31.5 µg/m^3^), and high NO_2_ (> 31.5 µg/m^3^). Both categorical and continuous air pollution exposure data were used in the data analyses.

### Definition of genetic risk score

We created a genetic risk score (GRS) for T2D using 102 single nucleotide polymorphisms (SNPs), which passed quality control, based on a previous study (Supplementary Table 1S) [18]. A weighted method was used to calculate the T2D GRS. We calculated the sum of the number of associated alleles (0, 1, or 2); each SNP was weighted by the strength of its association with T2D in a previous genome-wide association study [19]. The T2D GRSs showed a normal distribution (Supplementary Fig. 2S), and a higher T2D GRS indicated a higher genetic predisposition to T2D. The T2D GRS was then divided into tertiles to stratify individuals into high, intermediate, and low genetic risk categories. Detailed information about genotyping, imputation, and quality control in the UK Biobank study has been described previously [20].

### Ascertainment of type 2 diabetes

The prevalence and incidence of diabetes were assessed based on the UK Biobank algorithms for the diagnosis of diabetes [21]. Incident T2D was ascertained using hospital inpatient records containing data on admissions and diagnoses obtained from the Hospital Episode Statistics for England, Scottish Morbidity Record data for Scotland, and the Patient Episode Database for Wales [22]. Participants with T2D were defined by the International Classification of Diseases, 10th revision (ICD-10) code E11. Follow-up time was defined as the time from the date of attendance until the date of first diagnosis, February 28, 2017, for Scotland, or February 25, 2018, for Wales and England, whichever occurred first. Detailed information on the ascertainment of T2D is available online at http://content.digital.nhs.uk/services.

### Assessment of covariates

Potential confounders were selected according to the previously published literature [8, 23, 24]. We used the baseline touch screen questionnaire to assess several potential confounders: age, sex, race, education, household income, smoking status, alcohol consumption, body mass index (BMI), vegetable intake, fruit intake, family history of diabetes, comorbidities (hypertension, cardiovascular disease [CVD], depression, and cancer), genotyping chip and first 10 principal components of ancestry. BMI was calculated by dividing a participant’s weight by the square of his or her height in meters (kg/m^2^). Hypertension was defined as a self-reported history of hypertension, a systolic blood pressure ≥ 140 mmHg, a diastolic blood pressure ≥ 90 mmHg, or antihypertensive medication use. Supplementary Table 3S includes the coding of the variables under the assessment of the covariates. Details of these measurements are provided on the website of the UK Biobank (www.ukbiobank.ac.uk).

### Statistical analyses

Baseline characteristics of the participants are summarized across T2D status as means (standard deviations [SDs]) for continuous variables and numbers (percentages) for categorical variables. To impute the missing covariate values (all covariates had < 3% of the missing values), we used multiple imputation by chained equations using the R package “mice” to impute the missing covariate values [25]. Cox proportional hazard models were constructed to calculate hazard ratios (HRs) and 95% confidence intervals (CIs) for associations of PA, air pollution (PM_2.5_, PM_coarse_, PM_10_, or NO_2_), genetic risk, and their combination with incident T2D. The proportional hazards assumption, assessed using the Schoenfeld residuals technique [26], was satisfied.

We ran three models that included an increasing number of covariates: Model 1 included age and sex (men or women); Model 2 (multivariable-adjusted model) was adjusted as in Model 1 but also included race (white, Asian, black, Chinese, mixed, or other race), education (degree or no degree), household income (<£18,000, £18,000-£30,999, £31,000-£51,999, £52,000-£100,000, or >£100,000), smoking status (current, former, or never), alcohol consumption (current, former, or never), BMI, fruit and vegetable intake (< 2 or ≥ 2 servings per day), family history of diabetes (yes or no), hypertension (yes or no), CVD (yes or no), depression (yes or no), and cancer (yes or no); Model 3 was further mutually adjusted for PA and air pollution. PA and each air pollution variable were treated as continuous variables, and HRs were calculated per 600 MET-min/wk difference in PA, per 5 µg/m^3^ difference in PM_2.5_ and PM_coarse_, and per 10 µg/m^3^ difference in PM_10_ and NO_2_. Stratified analyses were conducted to examine the associations of PA in each air pollution stratum. To investigate the joint associations of PA and air pollution with T2D, the participants were then classified into 9 groups according to the categories of PA and each individual air pollutant with reference to the participants with low PA and high air pollutant exposure levels. For analyses of the genetic data, Model 3 was additionally adjusted for the genotyping chip and the first 10 principal components of ancestry. Interactions between PA, individual air pollutants and T2D GRS were assessed with the likelihood ratio test.

To examine the robustness of the primary findings, we conducted a series of sensitivity analyses. First, to examine the possibility of reverse causation bias, we excluded participants who developed T2D within the first two years of follow-up. Second, we excluded participants who had T2D-related diseases (CVD, cancer and hypertension) to eliminate potential comorbidity effects. Third, we restricted the analyses to participants with no missing covariate data. Fourth, employment status (working, retired, unemployed, other) of participants was further adjusted in the models. Fifth, participants were divided into 2 categories based on the WHO air quality guidelines for each air pollutant to examine the associations air pollution with incident T2D. Finally, we restricted the analyses to participants of European ancestry to test the association between T2D -GRS and incident T2D.

All analyses were performed using R software, version 4.0.2 (R Development Core Team, Vienna, Austria). A *p* value less than 0.05 was considered statistically significant in all analyses.

## Results

### Baseline characteristics

The baseline characteristics of the included participants (*n* = 359,153) are provided in Table 1. Overall, participants had a mean (SD) age of 56.3 (8.1) years, and 52.8% were women. Most participants (50.4%) performed a moderate volume of physical activity (600–3000 MET-min/wk). Participants who engaged in more PA were more likely to be former smokers and have a lower prevalence of hypertension, CVD, and depression. The mean (SD) annual average concentrations of PM_2.5_, PM_coarse_, PM_10_, and NO_2_ were 9.97 (1.06) µg/m^3^, 6.42 (0.90) µg/m^3^, 19.29 (1.96) µg/m^3^, and 29.14 (9.30) µg/m^3^, respectively. The pearson correlation coefficients of the air pollutants were shown in Supplementary Table 4S.

**Table 1:** Baseline characteristics of participants

Characteristic	Total	Physical activity		
		Low (<600 MET-min/wk)	Moderate (600-3000 MET-min/wk)	High (>3000 MET-min/wk)
*N*	359153 (100.0)	66735 (18.6)	180915 (50.4)	111503 (31.0)
Age, year	56.25 (8.12)	55.79 (7.92)	56.18 (8.13)	56.64 (8.21)
Female	189547 (52.8)	35324 (52.9)	98152 (54.3)	56071 (50.3)
Race				
White	331157 (92.2)	60720 (91.0)	166703 (92.1)	103734 (93.0)
Asian	15919 (4.4)	3420 (5.1)	8058 (4.5)	4441 (4.0)
Black	5676 (1.6)	1240 (1.9)	2876 (1.6)	1560 (1.4)
Chinese	1117 (0.3)	252 (0.4)	573 (0.3)	292 (0.3)
Mixed	2185 (0.6)	410 (0.6)	1114 (0.6)	661 (0.6)
Other ethnic group	3099 (0.9)	693 (1.0)	1591 (0.9)	815 (0.7)
Education				
Degree	124151 (34.6)	23095 (34.6)	69749 (38.6)	31307 (28.1)
No degree	235002 (65.4)	43640 (65.4)	111166 (61.4)	80196 (71.9)
Household income, £^a^				
<18,000	77131 (21.5)	14564 (21.8)	35734 (19.8)	26833 (24.1)
18,000 to 30,999	90720 (25.3)	15414 (23.1)	43778 (24.2)	31528 (28.3)
31,000 to 51,999	94909 (26.4)	17569 (26.3)	48150 (26.6)	29190 (26.2)
52,000 to 100,000	75748 (21.1)	15311 (22.9)	41228 (22.8)	19209 (17.2)
>100,000	20645 (5.7)	3877 (5.8)	12025 (6.6)	4743 (4.3)
BMI, mean (SD), kg/m^2^	27.22 (4.63)	28.42 (5.32)	27.07 (4.50)	26.75 (4.25)
Smoking status				
Never	197655 (55.0)	36160 (54.2)	101528 (56.1)	59967 (53.8)
Former	125343 (34.9)	22497 (33.7)	62993 (34.8)	39853 (35.7)
Current	36155 (10.1)	8078 (12.1)	16394 (9.1)	11683 (10.5)
Alcohol consumption				
Never	14176 (3.9)	3331 (5.0)	6648 (3.7)	4197 (3.8)
Former	11908 (3.3)	2628 (3.9)	5399 (3.0)	3881 (3.5)
Current	333069 (92.7)	60776 (91.1)	168868 (93.3)	103425 (92.8)
Vegetable intake, servings per day				
<2.0	119320 (33.2)	28566 (42.8)	60248 (33.3)	30506 (27.4)
≥2.0	239833 (66.8)	38169 (57.2)	120667 (66.7)	80997 (72.6)
Fruit intake, servings per day				
<2.0	126613 (35.3)	29395 (44.0)	62258 (34.4)	34960 (31.4)
≥2.0	232540 (64.7)	37340 (56.0)	118657 (65.6)	76543 (68.6)
Hypertension	87695 (24.4)	18170 (27.2)	43761 (24.2)	25764 (23.1)
Cancer	27211 (7.6)	27211 (7.6)	5226 (7.8)	13675 (7.6)
CVD	17953 (5.0)	4062 (6.1)	8460 (4.7)	5431 (4.9)
Depression	27744 (7.7)	6643 (10.0)	13334 (7.4)	7767 (7.0)
Family history of diabetes	61460 (17.1)	12220 (18.3)	30612 (16.9)	18628 (16.7)
PM_2.5_, µg/m^3^, mean (SD)	9.97 (1.06)	10.00 (1.04)	9.97 (1.06)	9.96 (1.06)
PM_coarse_, µg/m^3^, mean (SD)	6.42 (0.90)	6.43 (0.90)	6.42 (0.89)	6.42 (0.90)
PM_10_, µg/m^3^, mean (SD)	19.29 (1.96)	19.31 (1.90)	19.33 (1.98)	19.22 (1.96)
NO_2_, µg/m^3^, mean (SD)	29.14 (9.30)	29.21 (8.99)	29.32 (9.49)	28.80 (9.15)

^a^Data are presented as n (percent) unless otherwise indicated

### Associations of PA and air pollution with incident T2D

During a median of 8.9 (interquartile range: 8.2–9.5) years of follow-up, 13,706 T2D events were recorded. Table 2 shows the associations of PA and individual air pollution variables with incident T2D. We found that a higher level of PA was associated with a decreased risk of T2D after adjusting for a series of covariates, including air pollution. Compared with the low PA group, the moderate PA and high PA groups had adjusted HRs of 0.82 (95% CI, 0.79–0.86) and 0.73 (95% CI, 0.70–0.77), respectively. In contrast, higher air pollution levels were associated with an increased risk of T2D after adjusting for a series of covariates, including PA. Compared with the low air pollution group, the adjusted HRs for T2D in the moderate and high air pollution groups were 1.07 (95% CI,1.02–1.11) and 1.19 (95% CI,1.14–1.24) for PM_2.5_, 1.02 (95% CI, 0.98–1.07) and 1.06 (95% CI, 1.02–1.11) for PM_coarse_, 1.06 (95% CI, 1.02–1.11) and 1.13 (95% CI, 1.08–1.18) for PM_10_, and 1.08 (95% CI, 1.04–1.13) and 1.19 (95% CI, 1.14–1.24) for NO_2_, respectively. Moreover, we found significant trends for the associations of incident T2D across the categories of PA and all air pollution variables (Table 2).

**Table 2:** Association of physical activity or air pollution with risk of incident type 2 diabetes

Exposures	Events/ person-years	Model 1^a^		Model 2^b^		Model 3^c^	
		HR (95% CI)	*P* value	HR (95% CI)	*P* value	HR (95% CI)	*P* value
PA							
Low	5923/375968	1.00 (reference)	-	1.00 (reference)	-	1.00 (reference)	-
Moderate	3976/380077	0.60 (0.58-0.62)	<0.001	0.82 (0.79-0.86)	<0.001	0.82 (0.79-0.86)	<0.001
High	3807/379561	0.51 (0.49-0.54)	<0.001	0.73 (0.70-0.77)	<0.001	0.73 (0.70-0.77)	<0.001
Per 600 MET-min/week	-	0.96 (0.96-0.97)	<0.001	0.98 (0.98-0.99)	<0.001	0.98 (0.98-0.99)	<0.001
P for trend	-	-	<0.001	-	<0.001	-	<0.001
PM_2.5_							
Low	3793/377286	1.00 (reference)	-	1.00 (reference)	-	1.00 (reference)	-
Moderate	4533/378435	1.22 (1.17-1.27)	<0.001	1.07 (1.02-1.11)	0.003	1.07 (1.02-1.11)	<0.001
High	5380/379886	1.52 (1.46-1.58)	<0.001	1.19 (1.14-1.24)	<0.001	1.18 (1.14-1.24)	<0.001
Per 5µg/m^3^	-	2.36 (2.19-2.54)	<0.001	1.47 (1.36-1.59)	<0.001	1.47 (1.36-1.59)	<0.001
P for trend	-	-	<0.001	-	<0.001	-	<0.001
PM_coarse_							
Low	4168/378719	1.00 (reference)	-	1.00 (reference)	-	1.00 (reference)	-
Moderate	4621/379811	1.11 (1.07-1.16)	<0.001	1.02 (0.98-1.07)	0.263	1.02 (0.98-1.07)	0.263
High	4917/377076	1.20 (1.15-1.25)	<0.001	1.06 (1.02-1.11)	0.003	1.06 (1.02-1.11)	0.003
Per 5µg/m^3^	-	1.39 (1.27-1.52)	<0.001	1.15 (1.05-1.26)	0.002	1.15 (1.05-1.26)	0.003
P for trend	-	-	<0.001	-	<0.001	-	<0.001
PM_10_							
Low	4024/380363	1.00 (reference)	-	1.00 (reference)	-	1.00 (reference)	-
Moderate	4661/381420	1.19 (1.14-1.24)	<0.001	1.06 (1.02-1.11)	0.008	1.06 (1.02-1.11)	0.008
High	5021/373823	1.36 (1.30-1.41)	<0.001	1.13 (1.08-1.18)	<0.001	1.13 (1.08-1.18)	<0.001
Per 10µg/m^3^	-	1.98 (1.82-2.15)	<0.001	1.33 (1.21-1.45)	<0.001	1.33 (1.21-1.45)	<0.001
P for trend	-	-	<0.001	-	<0.001	-	<0.001
NO_2_							
Low	3820/383271	1.00 (reference)	-	1.00 (reference)	-	1.00 (reference)	-
Moderate	4632/382184	1.24 (1.19-1.30)	<0.001	1.08 (1.04-1.13)	<0.001	1.08 (1.04-1.13)	<0.001
High	5254/37150	1.52 (1.46-1.58)	<0.001	1.19 (1.14-1.24)	<0.001	1.19 (1.14-1.24)	<0.001
Per 10µg/m^3^	-	1.19 (1.17-1.21)	<0.001	1.08 (1.06-1.10)	<0.001	1.08 (1.06-1.10)	<0.001
P for trend	-	-	<0.001	-	<0.001	-	<0.001

*Abbreviation*: *CI* Confidence interval, *HR* Hazard ratio, *PA* Physical activity

^a^Model 1: adjusted for age, sex

^b^Model 2: adjusted for Model 1 and race, education, household income, smoking status, alcohol consumption, body mass index, fruit and vegetable intake, family history of diabetes, hypertension, cardiovascular disease, depression, and cancer

^c^Model 3: adjusted for Model 2 and air pollution or physical activity

### Joint effect of PA and air pollution on T2D

Table 3 indicates the associations between PA and incident T2D stratified by individual air pollution variables. Stratified analyses indicated that PA was inversely associated with the risk of T2D in each air pollution group. High levels of PA were associated with a 25-28% lower risk for T2D (HR between 0.72 and 0.75) than low PA levels at different levels of each air pollutant. No significant interactions between PA and air pollution were observed (Table 3 and Supplementary Table 5S; all *P*-interactions > 0.05).

**Table 3:** Risk of incident type 2 diabetes according to physical activity category within each of air pollution category

Air pollution	Low-PA		Moderate-PA		High-PA		P for trend	P-interaction^a^
	HR (95% CI)	*P* value	HR (95% CI)	*P* value	HR (95% CI)	*P* value		
PM_2.5_								0.359
Low	1.00 (reference)	-	0.82 (0.75-0.88)	<0.001	0.72 (0.66-0.79)	<0.001	<0.001	
Moderate	1.00 (reference)	-	0.85 (0.79-0.91)	<0.001	0.73 (0.67-0.79)	<0.001	<0.001	
High	1.00 (reference)	-	0.81 (0.76-0.86)	<0.001	0.75 (0.70-0.81)	<0.001	<0.001	
PM_coarse_								0.443
Low	1.00 (reference)	-	0.84 (0.78-0.91)	<0.001	0.73 (0.67-0.80)	<0.001	<0.001	
Moderate	1.00 (reference)	-	0.79 (0.74-0.85)	<0.001	0.73 (0.67-0.79)	<0.001	<0.001	
High	1.00 (reference)	-	0.79 (0.74-0.85)	<0.001	0.73 (0.67-0.79)	<0.001	<0.001	
PM_10_								0.879
Low	1.00 (reference)	-	0.82 (0.76-0.89)	<0.001	0.75 (0.68-0.81)	<0.001	<0.001	
Moderate	1.00 (reference)	-	0.82 (0.76-0.88)	<0.001	0.74 (0.68-0.80)	<0.001	<0.001	
High	1.00 (reference)	-	0.83 (0.78-0.89)	<0.001	0.73 (0.67-0.79)	<0.001	<0.001	
NO_2_								0.737
Low	1.00 (reference)	-	0.83 (0.76-0.9)	<0.001	0.75 (0.69-0.83)	<0.001	<0.001	
Moderate	1.00 (reference)	-	0.82 (0.76-0.88)	<0.001	0.72 (0.67-0.78)	<0.001	<0.001	
High	1.00 (reference)	-	0.83 (0.78-0.89)	<0.001	0.74 (0.68-0.80)	<0.001	<0.001	

Results obtained after adjusting age, sex race, education, household income, smoking status, alcohol consumption, body mass index, fruit and vegetable intake, family history of diabetes, hypertension, cardiovascular disease, depression, and cancer

*Abbreviation*: *CI* Confidence interval, *HR* Hazard ratio, *PA* Physical activity

^a^P-interaction describes the interactions between PA and air pollution. The interactions between PA and individual air pollutants were assessed with the likelihood ratio test by including an interaction term between the PA (low-, moderate-, and high-PA were coded as 0, 1 and 2 respectively) and the air pollution (low-, moderate-, and high-air pollution were coded as 0, 1 and 2 respectively) in the multivariable-adjusted model

**Fig. 1:**
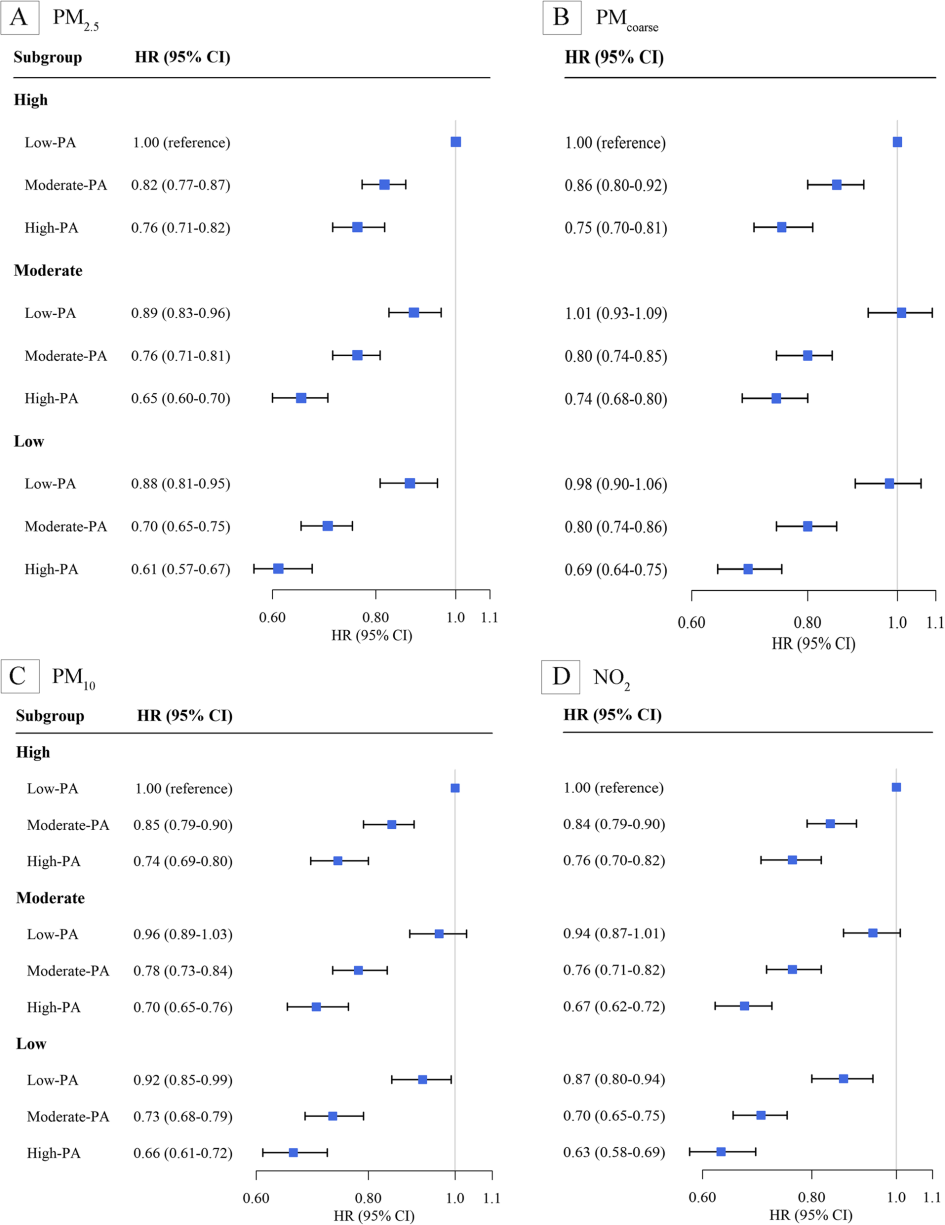
Joint associations of physical activity and PM_2.5_ (**A**), PM_10_ (**B**), PM_coarse_ (**C**), and NO_2_ (**D**) with the incidence of type 2 diabetes. Abbreviations: CI, confidence interval; HR, hazard ratio; PA, physical activity. The results were obtained after adjusting for age, sex, race, education, household income, smoking status, alcohol consumption, body mass index, fruit and vegetable intake, family history of diabetes, hypertension, cardiovascular disease, depression, and cancer

Figure 1 presents the joint associations of PA and air pollution with the risk of T2D. The analyses indicated that participants in the high PA and low air pollution groups had the lowest risk of T2D. Compared to participants in the low PA and high air pollution groups, the HR for T2D among participants with high PA in the low PM_2.5_ group was 0.61 (95% CI: 0.57–0.67), in the low PM_coarse_ group was 0.69 (95% CI: 0.64–0.75), in the low PM_10_ group was 0.66 (95% CI: 0.61–0.72), and in the low NO_2_ group was 0.63 (95% CI: 0.58–0.69). The inverse associations between PA and T2D in each air pollution stratum were generally consistent (all *P* for trend < 0.05).

The sensitivity analyses showed no substantial changes when we excluded participants who developed T2D during the first two years of follow-up (Supplementary Fig. 3S), those for whom covariate data were missing (Supplementary Fig. 4S), those who had T2D-related diseases (CVD, cancer and hypertension) (Supplementary Fig. 5S), further adjusted the employment status in the models (Supplementary Fig. 6S), or divided participants according to the WHO air quality guidelines (Supplementary Table 6S).

### Joint effect of PA or air pollution and T2D GRS on T2D

**Fig. 2:**
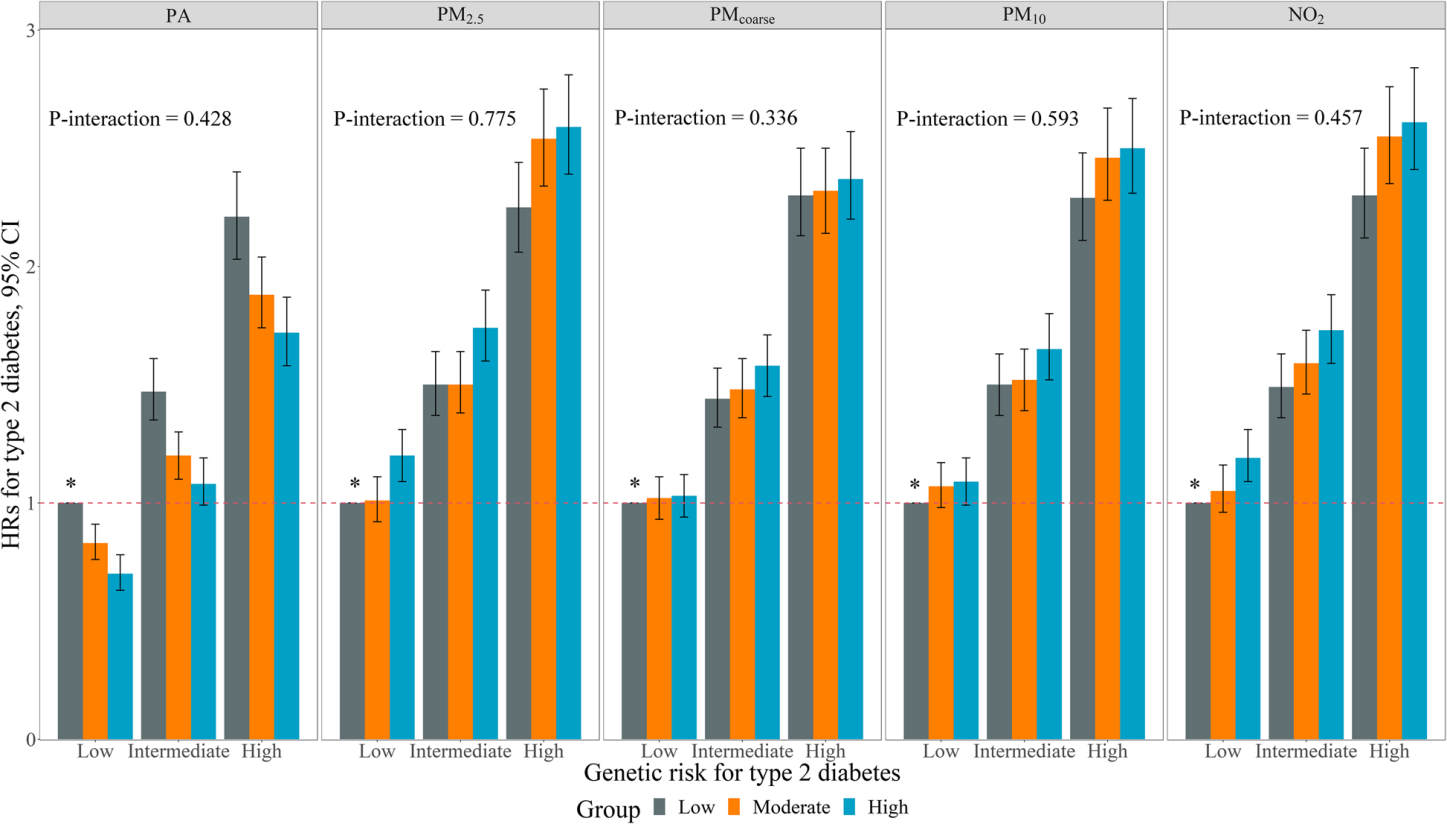
Joint associations of physical activity or air pollution and genetic risk with the incidence of type 2 diabetes. Abbreviations: CI, confidence interval; HR, hazard ratio. The results were obtained after adjusting for age, sex, race, education, household income, smoking status, alcohol consumption, body mass index, fruit and vegetable intake, family history of diabetes, hypertension, cardiovascular disease, depression, cancer, genotyping, the first 10 genetic principal components, and air pollution or physical activity. Individuals in the low PA or low air pollution group were used as the reference group (*). P-interaction describes the interactions between PA or air pollution and the genetic risk of type 2 diabetes. The interactions between PA or air pollution and T2D GRS were assessed with the likelihood ratio test by including an interaction term between PA (low, moderate, and high PA were coded as 0, 1 and 2, respectively) or air pollution (low, moderate, and high air pollution were coded as 0, 1 and 2, respectively) and T2D GRS (low, intermediate, and high PA were coded as 0, 1 and 2, respectively) in the multivariable-adjusted model

Genetic data were available for 351,185 participants in this study. In the multivariable-adjusted model, compared with participants with a low T2D GRS, those with an intermediate (HR: 1.45, 95% CI: 1.38–1.52) or high (HR: 2.20, 95% CI: 2.10–2.30) T2D GRS had an increased risk of T2D (Supplementary Table 7S). No substantial changes of the associations when we restricted the analyses to participants of European ancestry (Supplementary Table 7S). In the joint effect analyses, PA and each air pollution variable were significantly associated with the risk of T2D independent of T2D GRS (Fig. 2). There was no significant interaction between PA or each type of air pollutant and T2D GRS (all *P*-interactions > 0.05), indicating that the associations with PA and each type of air pollutant did not vary substantially on the basis of genetic risk.

## Discussion

In this large population-based cohort study involving 359,153 individuals, we identified the following important findings: (1) higher PA and lower air pollution exposure (PM_2.5_, PM_coarse_, PM_10_, and NO_2_) levels were associated with a lower risk of T2D after adjusting for a series of covariates, including a mutual adjustment for PA or air pollution; (2) the inverse association between PA and incident T2D generally remained stable among participants exposed to different levels of each air pollutant (all *P*-interactions > 0.05); and (3) there was no significant interaction between PA or air pollution and genetic risk (all *P*-interactions > 0.05), and higher PA and lower air pollution exposure levels were associated with a lower risk of T2D regardless of genetic risk.

Our significant finding of a low risk of developing T2D related to PA was consistent with existing evidence [27, 28]. Notably, a recent meta-analysis summarized 55 cohort studies and reported a 28% lower risk of T2D among participants with high PA levels than among participants with low PA levels [27]; similarly, our study indicated a 27% (HR: 0.73, 95% CI: 0.70–0.77) lower risk of T2D among participants with high PA levels than among those with low PA levels. Several mechanisms might explain the effect of PA on T2D. For example, PA might reduce the risk of T2D by increasing cardiorespiratory fitness, improving lipid levels and endothelial function [29], reducing glycosylated hemoglobin (HbA1c) levels and improving insulin sensitivity [30]. The activation of anti-inflammatory signaling pathways may be another potential mechanism underlying the effect of PA on T2D [31, 32]. In addition, we found that long-term exposure of individuals to air pollutants, including PM_2.5_, PM_coarse_, PM_10_, and NO_2_, was associated with a higher risk of T2D, providing further evidence of a positive association between long-term exposure to air pollution and T2D [5–7, 33, 34]. The adverse effects of air pollution on HbA1c and fasting glucose concentrations have been well documented in previous studies [33]. In addition, air pollution was associated with increased levels of systemic inflammation [35, 36] and oxidative stress [37], which may increase the risk of T2D [38, 39]. Overall, our study further supported the need to establish measures to increase PA and tackle air pollution, which might contribute to reducing the burden of diabetes.

Whether the health benefits of PA are moderated by long-term exposure to air pollution remains in dispute. Notably, a few studies found significant interactions between air pollution and PA, including evidence that air pollution counteracted the benefits of PA on asthma [40] and stroke [41]. In contrast, several studies revealed no significant interactions between PA and long-term exposure to air pollution on lung function/respiratory diseases [42, 43], hypertension [24], myocardial infarction [44], and mortality [45]. Other studies have also shown that although PA did not interact with air pollution, it counteracted the hazardous effect of air pollution on blood pressure [46] and atherosclerotic cardiovascular disease [47]. This discrepancy may be partially attributed to the differences in sample sizes, study durations, and the levels of air pollution in the study regions[24]. Moreover, recent studies found that the benefits of PA for diabetes did not interact with fine particulate matter and outweighed the risks related to ambient particulate matter exposure [8, 9]. Consistent with those findings, we found in the current study that the long-term benefits of PA with respect to T2D were not significantly moderated by exposure to air pollution. Furthermore, our finding that the long-term benefits of PA for T2D were not moderated by exposure to high levels of NO_2_ is novel. The current study, therefore, may indicate that the effects of long-term exposure to air pollution and PA on T2D are independent of each other, with the benefits of PA not being reduced by exposure to air pollution, including PM_2.5_, PM_coarse_, PM_10_, and NO_2_.

Interestingly, our study found that the associations of PA with T2D remained consistent among participants exposed to different individual air pollution levels, indicating that PA may decrease the risk of T2D, be it among people with relatively high or low levels of air pollution exposure. However, the exact reasons for the stable protective effect of PA on T2D regardless of air pollution have not been clarified. One potential hypothesis to explain this observation is that the additional air pollutants inhaled during PA account for only a small fraction of the total inhaled dose of air pollution [48] and, therefore, are not sufficient to increase the risk of T2D. It is also possible that health benefits due to increased PA levels generally outweigh the risks related to increases in inhaled air pollution doses during physical activity or exercise [49]. Thus, our study highlights an increase in PA as a potentially effective measure for the prevention of the incidence and progression of T2D, regardless of air pollution exposure.

Consistent with previous studies [50, 51], the T2D GRS was significantly positively associated with the risk of T2D in the current study. However, we did not observe a significant interaction between PA or air pollution and T2D GRS on the risk of T2D, suggesting that higher PA levels or reducing air pollution exposure may protect against T2D, regardless of the genetic risk profile.

### Strengths and limitations

The strengths of this study included a large sample size and outcome events, the inclusion of information on a wealth of potential confounders, highly accurate diabetes diagnoses by UK Biobank algorithms, confirmation of T2D events by medical record review, and uniform data collection protocols, which reduced measurement error and thus reduced biased estimates. Another major novelty of the current study is the examination of the interactions of PA and air pollution with genetic risk.

However, there were several limitations in this study that should be noted. First, we did not distinguish between indoor and outdoor PA, and indoor air pollution and relocation of participants during the study period were not considered in this study, which may contribute to exposure misclassification. Second, the assessment of exposure to air pollution relied on residence locations and did not completely reflect personal exposure. Given the inherent imprecision of the spatially derived exposure levels, the air pollution assessment was likely subject to nondifferential misclassification that may have attenuated our results. Third, air pollution exposure in our study was available at only certain periods in time. Although we made a reasonable assumption that the spatial contrast in air pollution exposure was relatively stable in the UK over these years, the possibility of exposure misclassification cannot be excluded. Fourth, our study was conducted in a low-pollution area. Further studies in areas with moderate and severe pollution are needed to examine the applicability of our findings. Fifth, although this study tried to identify incident T2D based on hospital inpatient records, there are no biomarkers (e.g., HbA1c and fasting blood glucose) to assess the status of T2D. Furthermore, in observational studies, the possibility of residual confounding factors due to imprecise measurements or unknown factors cannot be excluded. Therefore, although we carefully adjusted for various confounders in our analyses, the associations might have been affected by unknown factors. Finally, reverse causality might exist in our study, although the results remained unchanged when we excluded participants with outcome events that occurred during the first two years of follow-up.

## Conclusion

In summary, the results of this large-scale prospective cohort study showed that higher PA and lower air pollution levels were independently associated with a lower risk of T2D, and the benefits of PA for T2D generally remained stable among participants exposed to different levels of each air pollution variable, including PM_2.5_, PM_coarse_, PM_10_, and NO_2_. Our findings suggested that PA should be promoted to prevent T2D among people with both relatively high and low levels of air pollution exposure. Further studies are needed to validate our findings in regions with moderate and severe pollution levels.

## Supplementary Information

**Additional file 1: Table 1S.** Distribution of population characteristics in included individuals and the total population of UK Biobank. **Table 2S.** Single-nucleotide polymorphisms used to build the genetic risk score for type 2 diabetes. **Table 3S.** The coding of the variables under assessment of the covariates in the UK Biobank study. **Table 4S.** Pearson correlation coefficients among the five air pollutants. **Table 5S.** Risk of incident type 2 diabetes according to air pollution within each of physical activity category. **Table 6S.** Association of air pollution with risk of incident type 2 diabetes according to WHO air quality guidelines. **Table 7S.** Hazard ratios for type 2 diabetes across tertiles categories of type 2 diabetes genetic risk score. **Figure 1S.** Flow chart of participants enrolment. **Figure 2S.** Distribution of the polygenic risk score for type 2 diabetes. Figure 3S. Joint associations of physical activity and air pollutants with incidence of type 2 diabetes after excluding participants with type 2 diabetes within 2 years of baseline. **Figure 4S.** Joint associations of physical activity and air pollutants with incidence of type 2 diabetes after excluding participants with missing data for covariates.**Figure 5S.** Joint associations of physical activity and air pollutants with incidence of type 2 diabetes after excluding participants who had type 2 diabetes related diseases. **Figure 6S.** Joint associations of physical activity and air pollutants with incidence of type 2 diabetes after adjusting the employment status. **Figure 7S.** Joint associations of physical activity or air pollution and genetic risk with the incidence of type 2 diabetes after excluding participants of non-European ancestry.

## Data Availability

The UK Biobank data are available from the UK Biobank upon request (www.ukbiobank.ac.uk/).

## Abbreviations

BMI: Body mass index
CIs: Confidence intervals
CVD: Cardiovascular disease
ESCAPE: European Study of Cohorts and Air Pollution Effects
GRS: Genetic risk score
HbA1c: Glycosylated hemoglobin
HRs: Hazard ratios
ICD-10: International Classification of Diseases, 10th revision
IPAQ: International Physical Activity Questionnaire
LUR: Land use regression
MET: Metabolic equivalent of task
NO2: Nitrogen dioxide
PA: Physical activity
PM: Particulate matter
SD: Standard deviation
SNPs: Single nucleotide polymorphisms
T2D: Type 2 diabetes
