# Supplementary material for: Association of physical activity and air pollution exposure with the risk of type 2 diabetes: a large population-based prospective cohort study
Source: Environ Health. 2022 Nov 6;21:106. doi: 10.1186/s12940-022-00922-3 (PMC9639290; doi:10.1186/s12940-022-00922-3)
Supplement: Supplementary file 1 — Additional file 1: Table 1S. Distribution of population characteristics in included individuals and the total population of UK Biobank. Table 2S. Single-nucleotide polymorphisms used to build the genetic risk score for type 2 diabetes. Table 3S. The coding of the variables under assessment of the covariates in the UK Biobank study. Table 4S. Pearson correlation coefficients among the five air pollutants. Table 5S. Risk of incident type 2 diabetes according to air pollution within each of physical activity category. Table 6S. Association of air pollution with risk of incident type 2 diabetes according to WHO air quality guidelines. Table 7S. Hazard ratios for type 2 diabetes across tertiles categories of type 2 diabetes genetic risk score. Figure 1S. Flow chart of participants enrolment. Figure 2S. Distribution of the polygenic risk score for type 2 diabetes. Figure 3S. Joint associations of physical activity and air pollutants with incidence of type 2 diabetes after excluding participants with type 2 diabetes within 2 years of baseline. Figure 4S. Joint associations of physical activity and air pollutants with incidence of type 2 diabetes after excluding participants with missing data for covariates.Figure 5S. Joint associations of physical activity and air pollutants with incidence of type 2 diabetes after excluding participants who had type 2 diabetes related diseases. Figure 6S. Joint associations of physical activity and air pollutants with incidence of type 2 diabetes after adjusting the employment status. Figure 7S. Joint associations of physical activity or air pollution and genetic risk with the incidence of type 2 diabetes after excluding participants of non-European ancestry. [file 12940_2022_922_MOESM1_ESM.docx]

**Supplementary Online Content**

[**Table 1S.** Distribution of population characteristics in included individuals and the total population of UK Biobank.](#_Toc19112)

[**Table 2S.** Single-nucleotide polymorphisms used to build the genetic risk score for type 2 diabetes.](#_Toc10626)

[**Table 3S.** The coding of the variables under assessment of the covariates in the UK Biobank study.](#_Toc13351)

[**Table 4S.**](#_Toc4238) Pearson correlation coefficients among the five air pollutants.

[**Table 5S.** Risk of incident type 2 diabetes according to air pollution within each of physical activity category.](#_Toc28810)

[**Table 6S.** Association of air pollution with risk of incident type 2 diabetes according to WHO air quality guidelines.](#_Toc29296)

[**Table 7S.** Hazard ratios for type 2 diabetes across tertiles categories of type 2 diabetes genetic risk score.](#_Toc17117)

[**Figure 1S.** Flow chart of participants enrolment.](#_Toc23593)

[**Figure 2S.** Distribution of the polygenic risk score for type 2 diabetes.](#_Toc2489)

[**Figure 3S.** Joint associations of physical activity and air pollutants with incidence of type 2 diabetes after excluding participants with type 2 diabetes within 2 years of baseline.](#_Toc2339)

[**Figure 4S.** Joint associations of physical activity and air pollutants with incidence of type 2 diabetes after excluding participants with missing data for covariates.](#_Toc21833)

[**Figure 5S.** Joint associations of physical activity and air pollutants with incidence of type 2 diabetes after excluding participants who had type 2 diabetes related diseases.](#_Toc10648)

[**Figure 6S.** Joint associations of physical activity and air pollutants with incidence of type 2 diabetes after adjusting the employment status.](#_Toc31386)

[**Figure 7S.** Joint associations of physical activity or air pollution and genetic risk with the incidence of type 2 diabetes after excluding participants of non-European ancestry.](#_Toc30007)

# Table 1S. Distribution of population characteristics in included individuals and the total population of UK Biobank.

| **Characteristic** | **Included individuals** | **Total population of UK Biobank** |
| --- | --- | --- |
| *N* | 359153 | 501237 |
| Age, year | 56.25 (8.12) | 56.53 (8.09) |
| Female | 189547 (52.8) | 272709 (54.4) |
| Race |  |  |
| White | 331157 (92.2) | 458393 (91.5) |
| Asian | 15919 (4.4) | 23020 (4.6) |
| Black | 5676 (1.6) | 8024 (1.6) |
| Chinese | 1117 (0.3) | 1568 (0.3) |
| Mixed | 2185 (0.6) | 2944 (0.6) |
| Other ethnic group | 3099 (0.9) | 4531 (0.9) |
| Education |  |  |
| Degree | 124151 (34.6) | 160561 (32.0) |
| No degree | 235002 (65.4) | 340676 (68.0) |
| Household income, £^*^ |  |  |
| <18,000 | 77131 (21.5) | 97020 (19.4) |
| 18,000 to 30,999 | 90720 (25.3) | 107958 (21.5) |
| 31,000 to 51,999 | 94909 (26.4) | 110509 (22.0) |
| 52,000 to 100,000 | 75748 (21.1) | 85980 (17.2) |
| >100,000 | 20645 (5.7) |  |
| BMI, mean (SD), kg/m^2^ | 27.22 (4.63) | 27.43 (4.80) |
| Smoking status |  |  |
| Never | 197655 (55.0) | 272884 (54.4) |
| Former | 125343 (34.9) | 172597 (34.4) |
| Current | 36155 (10.1) | 52827 (10.5) |
| Alcohol consumption |  |  |
| Never | 14176 (3.9) | 459219 (91.6) |
| Former | 11908 (3.3) | 22322 (4.5) |
| Current | 333069 (92.7) | 18057 (3.6) |
| Vegetable intake, servings per day |  |  |
| <2.0 | 119320 (33.2) | 176832 (35.3) |
| ≥2.0 | 239833 (66.8) | 321185 (64.1) |
| Fruit intake, servings per day |  |  |
| <2.0 | 126613 (35.3) | 169424 (33.8) |
| ≥2.0 | 232540 (64.7) | 320900 (64.0) |
| Hypertension | 87695 (24.4) | 282381 (56.3) |
| Cancer | 27211 (7.6) | 38514 (7.7) |
| CVD | 17953 (5.0) | 32493 (6.5) |
| Depression | 27744 (7.7) | 38595 (7.7) |
| Family history of diabetes | 61460 (17.1) | 87243 (17.4) |

Abbreviations: BMI, body mass index; CVD, cardiovascular disease; PA, physical activity; SD, standard deviation; T2D, type 2 diabetes;

Data are presented as n (percent) unless otherwise indicated.

^*^£18,000 = €21,489; $23,253.

# Table 2S. Single-nucleotide polymorphisms used to build the genetic risk score for type 2 diabetes.

| **Chromosome** | **Locus** | **SNP** | **Effect Allele** | **Reference Allele** | **EAF** | **OR** |
| --- | --- | --- | --- | --- | --- | --- |
| 1 | *MACF1* | rs3768321 | T | G | 0.1926 | 1.08 |
| 1 | *FAF1* | rs12031920 | T | A | 0.5555 | 1.05 |
| 1 | *NOTCH2* | rs406767 | C | T | 0.0900 | 1.14 |
| 1 | *ATP8B2* | rs67156297 | A | G | 0.2538 | 1.03 |
| 1 | *PROX1* | rs340874 | C | T | 0.5502 | 1.07 |
| 2 | *GCKR* | rs145819220 | G | C | 0.0115 | 1.26 |
| 2 | *THADA* | rs6757251 | C | T | 0.9011 | 1.14 |
| 2 | *ASB3* | rs9309245 | G | C | 0.3369 | 1.01 |
| 2 | *CCDC85A* | rs1116357 | G | A | 0.5111 | 1.01 |
| 2 | *BCL11A* | rs10193447 | T | C | 0.5979 | 1.07 |
| 2 | *TMEM163* | rs6723108 | T | G | 0.4990 | 1.02 |
| 2 | *RBM43/RND3* | rs7560163 | C | G | 0.9967 | 1.17 |
| 2 | *RBMS1* | rs1563575 | A | G | 0.7420 | 1.07 |
| 2 | *GRB14* | rs28584669 | T | C | 0.8333 | 1.05 |
| 2 | *IRS1* | rs2972156 | G | C | 0.6143 | 1.08 |
| 2 | *DNER* | rs1861612 | A | G | 0.5430 | 1.02 |
| 3 | *PPARG* | rs11712037 | C | G | 0.8746 | 1.14 |
| 3 | *UBE2E2* | rs35352848 | T | C | 0.7771 | 1.09 |
| 3 | *PSMD6* | rs79819696 | A | G | 0.004 | 1.39 |
| 3 | *ADAMTS9* | rs7428936 | T | C | 0.5905 | 1.07 |
| 3 | *ADCY5* | rs11708067 | A | G | 0.7871 | 1.12 |
| 3 | *IGF2BP2* | rs4402960 | T | G | 0.3056 | 1.15 |
| 3 | *ST6GAL1* | rs9820223 | C | T | 0.3829 | 1.06 |
| 3 | *LPP* | rs6777684 | G | A | 0.6057 | 1.05 |
| 4 | *MAEA* | rs1531583 | T | G | 0.0540 | 1.15 |
| 4 | *WFS1* | rs3821943 | T | C | 0.5351 | 1.10 |
| 4 | *TMEM154* | rs7660590 | C | T | 0.7162 | 1.06 |
| 4 | *ACSL1* | rs60780116 | T | C | 0.8354 | 1.09 |
| 5 | *ARL15* | rs11747901 | G | C | 0.1884 | 1.07 |
| 5 | *ANKRD55* | rs9687833 | A | G | 0.1865 | 1.10 |
| 5 | *ANKRD55* | rs173964 | G | A | 0.7436 | 1.06 |
| 5 | *ZBED3* | rs6453287 | C | A | 0.3041 | 1.07 |
| 5 | *PAM* | rs78408340 | G | C | 0.0094 | 1.40 |
| 5 | *PAM* | rs74944275 | T | C | 0.0414 | 1.16 |
| 6 | *SSR1/RREB1* | rs6923241 | C | T | 0.7118 | 1.07 |
| 6 | *CDKAL1* | rs7451008 | C | T | 0.2606 | 1.19 |
| 6 | *POU5F1/TCF19* | rs115321690 | G | A | 0.6776 | 1.07 |
| 6 | *HLA-B* | rs2244020 | G | A | 0.3668 | 1.02 |
| 6 | *HLA-DQA1* | rs9271774 | C | A | 0.7416 | 1.10 |
| 6 | *ZFAND3* | rs143308245 | T | A | 0.0030 | 2.02 |
| 6 | *KCNK16* | rs139514607 | T | C | 0.0033 | 1.48 |
| 6 | *CENPW* | rs11759026 | G | A | 0.2371 | 1.10 |
| 6 | *SLC35D3* | rs6918311 | A | G | 0.5284 | 1.07 |
| 7 | *DGKB* | rs10276674 | C | T | 0.1981 | 1.09 |
| 7 | *DGKB* | rs10238625 | A | G | 0.5397 | 1.07 |
| 7 | *JAZF1* | rs1635852 | T | C | 0.5016 | 1.10 |
| 7 | *GCK* | rs878521 | A | G | 0.2400 | 1.05 |
| 7 | *PAX4* | rs10229583 | G | A | 0.7472 | 1.04 |
| 7 | *GCC1* | rs73455744 | A | G | 0.9970 | 1.93 |
| 7 | *MIR129-LEP* | rs791595 | G | A | 0.8254 | 1.01 |
| 7 | *KLF14* | rs10954284 | T | A | 0.5017 | 1.06 |
| 7 | *MNX1* | rs1182436 | C | T | 0.7977 | 1.08 |
| 8 | *ANK1* | rs516946 | C | T | 0.7750 | 1.08 |
| 8 | *TP53INP1* | rs4734285 | T | C | 0.6200 | 1.06 |
| 8 | *TP53INP1* | rs11786613 | C | A | 0.0317 | 1.21 |
| 8 | *SLC30A8* | rs3802177 | G | A | 0.6774 | 1.12 |
| 9 | *GLIS3* | rs10758593 | A | G | 0.4129 | 1.05 |
| 9 | *PTPRD* | rs186838848 | T | C | 0.0063 | 1.46 |
| 9 | *CDKN2A/B* | rs10965248 | T | C | 0.8191 | 1.15 |
| 9 | *CDKN2A/B* | rs10965223 | A | G | 0.5918 | 1.08 |
| 9 | *CDKN2A/B* | rs10757282 | C | T | 0.4362 | 1.04 |
| 9 | *DMRTA1* | rs1575972 | T | A | 0.9663 | 1.13 |
| 9 | *TLE4* | rs13301067 | G | A | 0.9236 | 1.11 |
| 9 | *TLE1* | rs9410573 | T | C | 0.5986 | 1.08 |
| 9 | *ABO* | rs635634 | T | C | 0.1800 | 1.08 |
| 9 | *GPSM1* | rs11787792 | A | G | 0.6735 | 1.04 |
| 10 | *CDC123/CAMK1D* | rs11257659 | T | C | 0.2293 | 1.08 |
| 10 | *VPS26A* | rs10998572 | C | A | 0.9345 | 1.09 |
| 10 | *ZMIZ1* | rs810517 | C | T | 0.5146 | 1.09 |
| 10 | *HHEX/IDE* | rs11187140 | G | A | 0.6222 | 1.14 |
| 10 | *TCF7L2* | rs7903146 | T | C | 0.2892 | 1.34 |
| 10 | *GRK5* | rs10886471 | C | T | 0.5046 | 1.02 |
| 10 | *PLEKHA1* | rs2292626 | C | T | 0.5038 | 1.09 |
| 11 | *DUSP8* | rs2334499 | T | C | 0.4154 | 1.05 |
| 11 | *INS-IGF2* | rs11564732 | T | C | 0.0246 | 1.02 |
| 11 | *MIR4686* | rs7107784 | G | A | 0.2828 | 1.02 |
| 11 | *KCNQ1* | rs756852 | G | A | 0.5975 | 1.09 |
| 11 | *KCNQ1* | rs231360 | T | C | 0.4069 | 1.08 |
| 11 | *KCNQ1* | rs233449 | G | A | 0.7260 | 1.09 |
| 11 | *KCNQ1* | rs2237897 | C | T | 0.9456 | 1.25 |
| 11 | *KCNQ1* | rs191294997 | G | A | 0.1426 | 1.06 |
| 11 | *KCNQ1* | rs441613 | C | T | 0.6331 | 1.06 |
| 11 | *KCNJ11* | rs5219 | T | C | 0.3828 | 1.07 |
| 11 | *HSD17B12* | rs1061810 | A | C | 0.2785 | 1.08 |
| 11 | *MAP3K11* | rs111669836 | A | T | 0.2485 | 1.07 |
| 11 | *ARAP1 (CENTD2)* | rs76550717 | A | G | 0.8299 | 1.10 |
| 11 | *MTNR1B* | rs10830963 | G | C | 0.2661 | 1.08 |
| 12 | *CCND2* | rs11063018 | C | T | 0.1902 | 1.09 |
| 12 | *CCND2* | rs188827514 | A | G | 0.9944 | 1.64 |
| 12 | *CCND2* | rs4238013 | C | T | 0.2001 | 1.10 |
| 12 | *KLHDC5* | rs7953190 | T | C | 0.8031 | 1.08 |
| 12 | *FAM60A* | rs147538848 | G | A | 0.9900 | 1.12 |
| 12 | *HMGA2* | rs2258238 | T | A | 0.1002 | 1.11 |
| 12 | *TSPAN8/LGR5* | rs6581998 | C | T | 0.2707 | 1.06 |
| 12 | *HNF1A (TCF1)* | rs56348580 | G | C | 0.6831 | 1.08 |
| 12 | *MPHOSPH9* | rs2851437 | A | C | 0.7189 | 1.07 |
| 13 | *SGCG* | rs9552911 | A | G | 0.0075 | 1.25 |
| 13 | *TBC1D4* | rs7330796 | C | T | 0.1136 | 1.02 |
| 13 | *SPRY2* | rs11616380 | G | T | 0.7148 | 1.09 |
| 14 | *NRXN3* | rs10146997 | G | A | 0.2118 | 1.07 |
| 15 | *RASGRP1* | rs7403531 | T | C | 0.213 | 1.04 |
| 15 | *INAFM2* | rs67839313 | C | T | 0.1127 | 1.03 |
| 15 | *C2CD4A* | rs4774420 | C | T | 0.7024 | 1.08 |
| 15 | *HMG20A* | rs952471 | G | C | 0.6869 | 1.08 |
| 15 | *ZFAND6* | rs62006309 | A | G | 0.5226 | 1.05 |
| 15 | *AP3S2* | rs62023387 | C | A | 0.1834 | 1.07 |
| 15 | *PRC1* | rs12595616 | C | T | 0.3668 | 1.07 |
| 16 | *FTO* | rs1558902 | A | T | 0.4155 | 1.13 |
| 16 | *BCAR1* | rs8056814 | G | A | 0.9168 | 1.16 |
| 16 | *CMIP* | rs2925979 | T | C | 0.2977 | 1.08 |
| 17 | *SRR* | rs9911305 | A | G | 0.7016 | 1.05 |
| 17 | *ZZEF1* | rs7224685 | T | G | 0.3043 | 1.07 |
| 17 | *SLC16A11/A13* | rs13342692 | C | T | 0.0147 | 1.07 |
| 17 | *GLP2R* | rs78761021 | G | A | 0.3414 | 1.07 |
| 17 | *HNF1B (TCF2)* | rs757209 | G | A | 0.5780 | 1.09 |
| 17 | *GIP* | rs79349575 | A | T | 0.5061 | 1.07 |
| 18 | *LAMA1* | rs7234111 | C | T | 0.3640 | 1.06 |
| 18 | *MC4R* | rs1942880 | T | C | 0.3323 | 1.07 |
| 18 | *MC4R* | rs79851087 | A | G | 0.9721 | 1.19 |
| 18 | *BCL2A* | rs12454712 | T | C | 0.6186 | 1.05 |
| 19 | *CILP2* | rs58489806 | T | C | 0.0913 | 1.09 |
| 19 | *PEPD* | rs139990642 | A | G | 0.0111 | 1.25 |
| 19 | *APOE* | rs429358 | T | C | 0.8475 | 1.13 |
| 19 | *GIPR* | rs55864746 | A | G | 0.3085 | 1.07 |
| 20 | *HNF4A* | rs12625671 | C | T | 0.1091 | 1.09 |
| 20 | *HNF4A* | rs1800961 | T | C | 0.0374 | 1.17 |
| 22 | *MTMR3/HORMAD2* | rs2023681 | G | A | 0.8878 | 1.13 |

Abbreviation: EAF, Effect Allele frequency; OR, odd ratio; SNP, Single nucleotide polymorphism.

# Table 3S. The coding of the variables under assessment of the covariates in the UK Biobank study.

| **Covariates** | **Field IDs** |
| --- | --- |
| Age | 21022 |
| Sex | 31 |
| Race | 21000 |
| Education | 6138 |
| Household income | 738 |
| BMI | 21001 |
| Smoking status | 20116 |
| Alcohol consumption | 20117 |
| Vegetable intake | 1289 |
| Fruit intake | 1309 |
| Hypertension | 4080, 4079, 41270, and 20003 |
| Cancer | 20002 |
| CVD | 41270 |
| Depression | 20126 |
| Family history of diabetes | 20107 and 20110 |

Abbreviations: BMI, body mass index; CVD, cardiovascular disease.

# Table 4S. Pearson correlation coefficients among the five air pollutants.

| Air pollutants | PM_2.5_ | PM_coarse_ | PM_10_ | NO_2_ |
| --- | --- | --- | --- | --- |
| PM_2.5_ | 1.00 | - | - | - |
| PM_coarse_ | 0.22^*^ | 1.00 | - | - |
| PM_10_ | 0.65^*^ | 0.53^*^ | 1.00 | - |
| NO_2_ | 0.73^*^ | 0.18^*^ | 0.80^*^ | 1.00 |

^*^ *P*<0.001

# Table 5S. Risk of incident type 2 diabetes according to air pollution within each of physical activity category.

| **Air pollution** | **Low-PA** | |  | **Moderate-PA** | |  | **High-PA** | | **P-interaction^a^** |
| --- | --- | --- | --- | --- | --- | --- | --- | --- | --- |
|  | **HR (95% CI)** | **P value** |  | **HR (95% CI)** | **P value** |  | **HR (95% CI)** | **P value** |  |
| PM_2.5_ (Per 5 µg/m^3^) | 1.41 (1.18-1.67) | <0.001 |  | 1.49 (1.29-1.72) | <0.001 |  | 1.43 (1.27-1.61) | <0.001 | 0.495 |
| PM_coarse_ (Per 5 µg/m^3^) | 1.10 (1.00-1.20) | 0.048 |  | 1.20 (1.01-1.42) | <0.001 |  | 1.15 (1.01-1.32) | 0.013 | 0.408 |
| PM_10_ (Per 10 µg/m^3^) | 1.28 (1.05-1.57) | 0.051 |  | 1.34 (1.14-1.58) | <0.001 |  | 1.29 (1.14-1.48) | <0.001 | 0.711 |
| NO_2_ (Per 10 µg/m^3^) | 1.08 (1.03-1.12) | <0.001 |  | 1.09 (1.06-1.13) | <0.001 |  | 1.07 (1.04-1.10) | <0.001 | 0.694 |

Abbreviation: CI, confidence interval; HR, hazard ratio; PA, physical activity.

Results obtained after adjusting age, sex race, education, household income, smoking status, alcohol consumption, body mass index, fruit and vegetable intake, family history of diabetes, hypertension, cardiovascular disease, depression, and cancer.

^a^P-interaction describes the interactions between PA and air pollution.

# Table 6S. Association of air pollution with risk of incident type 2 diabetes according to WHO air quality guidelines.

| **Air pollution** | HR (95% CI) | P value |
| --- | --- | --- |
| PM_2.5_ |  |  |
| ≤5 μg/m^3^ | 1.00 (reference) | - |
| >5 μg/m^3^ | 1.11 (1.08-1.15) | <0.001 |
| PM_10_ |  |  |
| ≤15 μg/m^3^ | 1.00 (reference) | - |
| >15 μg/m^3^ | 1.09 (1.05-1.13) | <0.001 |
| NO_2_ |  |  |
| ≤10 μg/m^3^ | 1.00 (reference) | - |
| >10 μg/m^3^ | 1.10 (1.05-1.16) | <0.001 |

Abbreviation: CI, confidence interval; HR, hazard ratio.

Results obtained after adjusting age, sex, race, education, household income, smoking status, alcohol consumption, body mass index, fruit and vegetable intake, family history of diabetes, hypertension, cardiovascular disease, depression, and cancer

.

# Table 7S. Hazard ratios for type 2 diabetes across tertiles categories of type 2 diabetes genetic risk score.

|  | **Low genetic risk** | **Intermediate genetic risk** | **High genetic risk** | **P for trend** |
| --- | --- | --- | --- | --- |
| T2D Events | 2830 | 4172 | 6348 |  |
| Model 1 | 1.00 (reference) | 1.46 (1.40-1.54) | 2.21 (2.11-2.31) | <0.001 |
| Model 2 | 1.00 (reference) | 1.45 (1.38-1.52) | 2.20 (2.10-2.30) | <0.001 |

Model 1: Results were adjusted for age, sex, genotyping, and the first 10 genetic principal component.

Model 2: Results were adjusted for age, sex race, education, household income, smoking status, alcohol consumption, BMI, and fruit and vegetable intake, family history of diabetes, hypertension, CVD, depression, cancer, genotyping, the first 10 genetic principal component, and air pollution or physical activity.


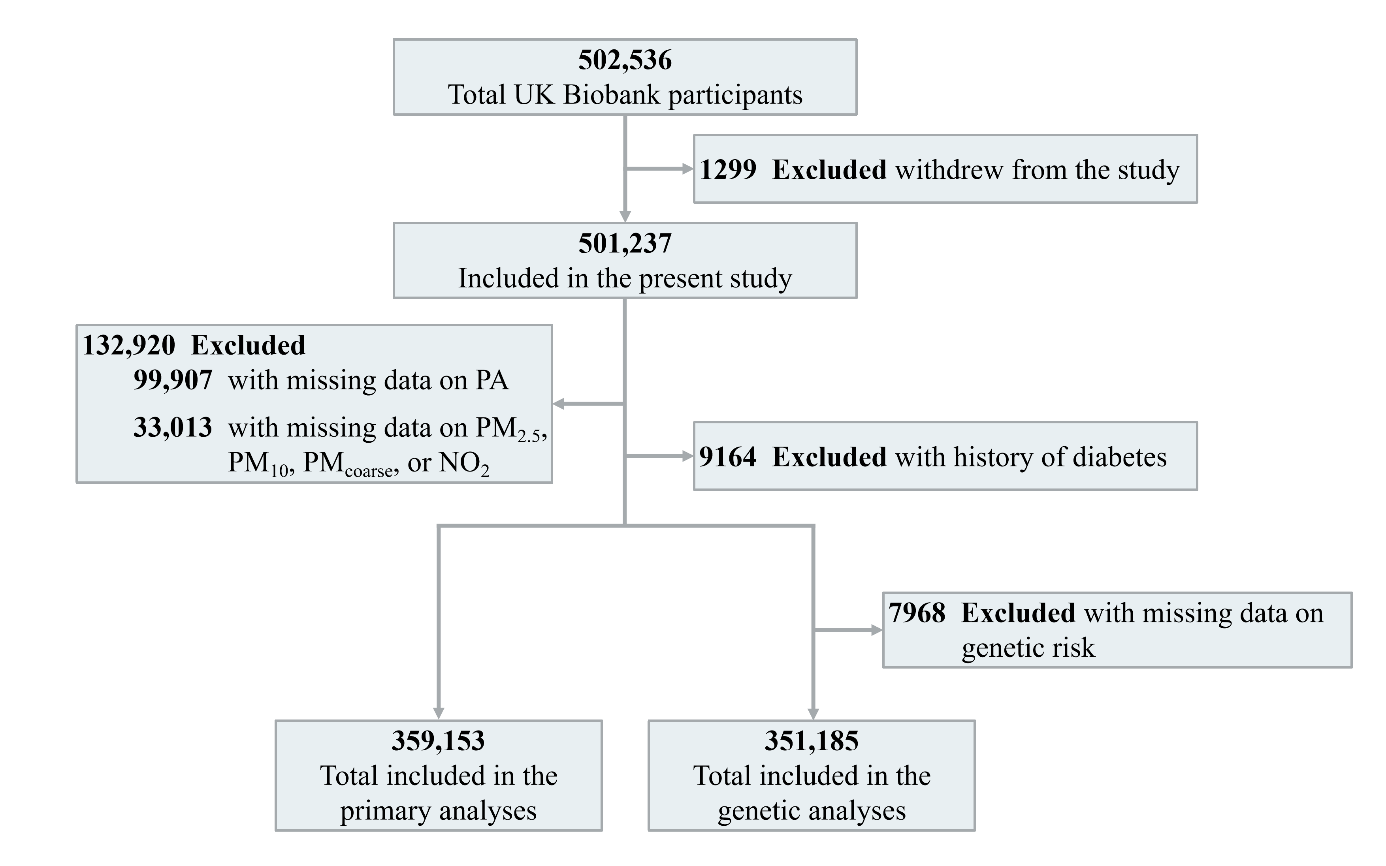


# Figure 1S. Flow chart of participants enrolment.

Abbreviation: PA, physical activity.


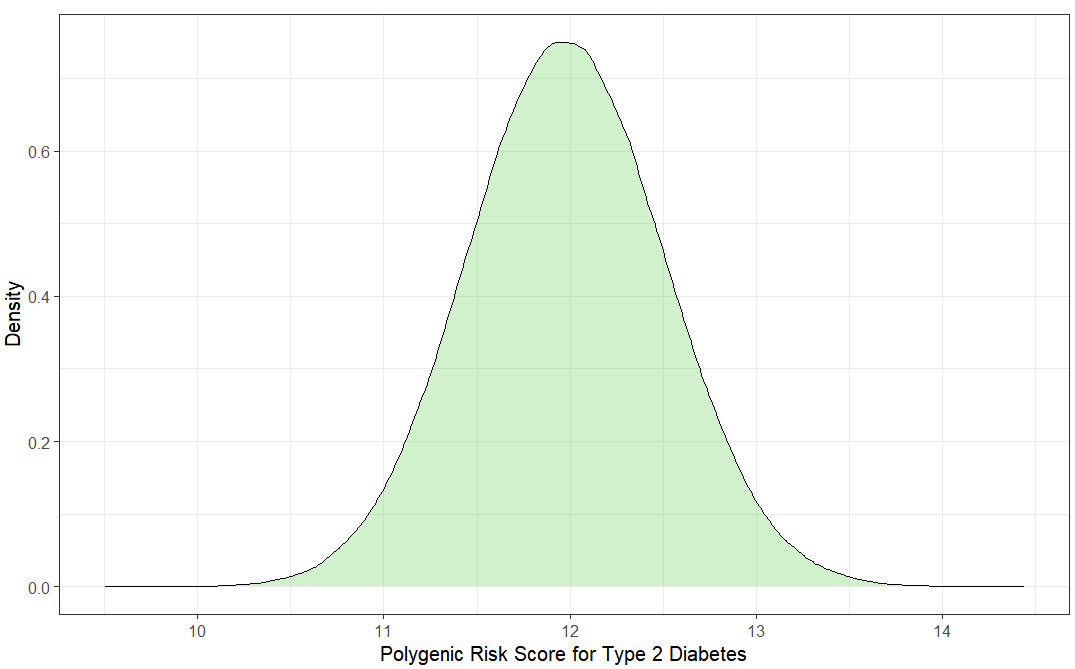


# Figure 2S. Distribution of the polygenic risk score for type 2 diabetes.


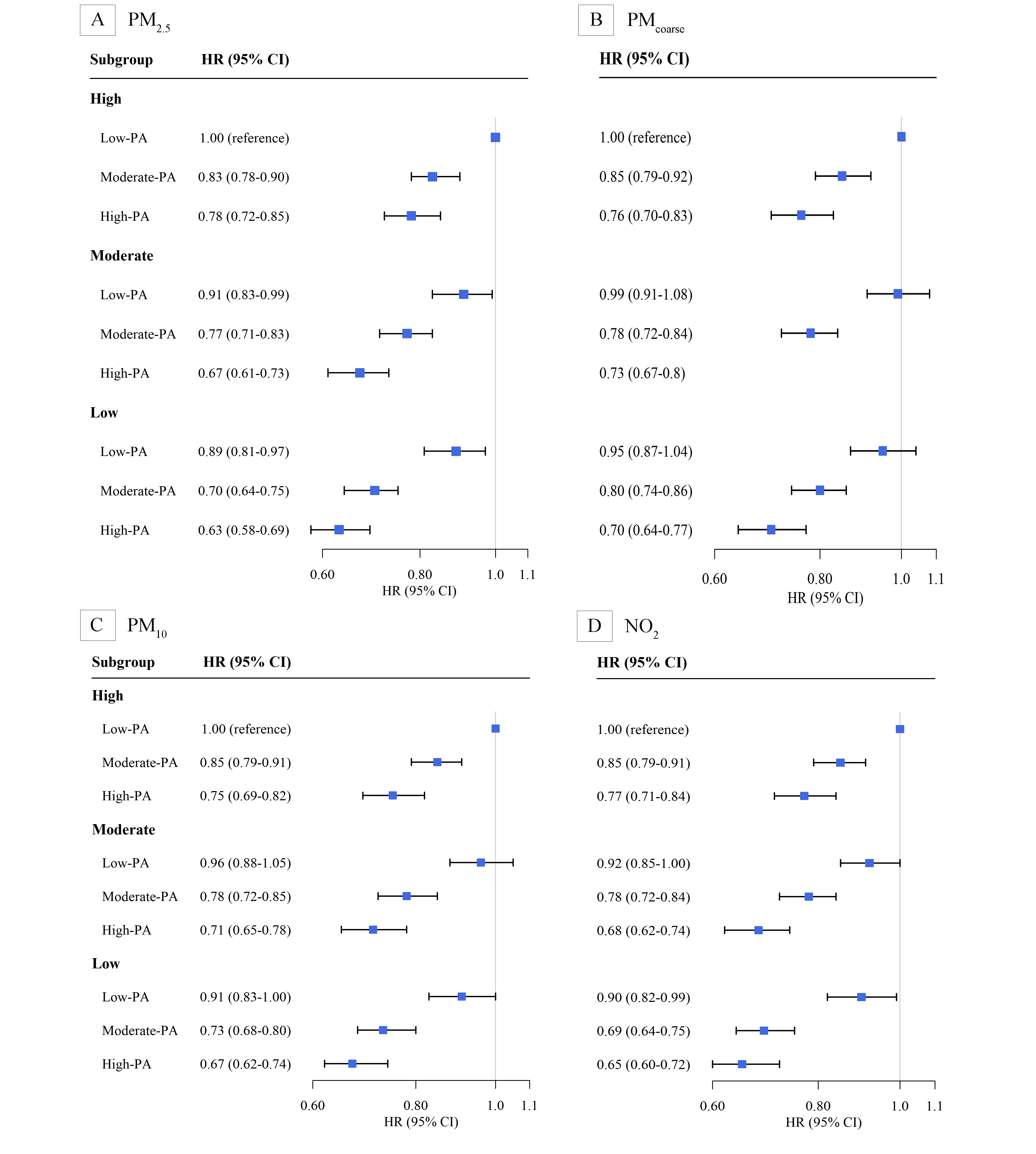


# Figure 3S. Joint associations of physical activity and air pollutants with incidence of type 2 diabetes after excluding participants with type 2 diabetes within 2 years of baseline.

Abbreviation: CI, confidence interval; HR, hazard ratio. Results obtained after adjusting age, sex race, education, household income, smoking status, alcohol consumption, BMI, and fruit and vegetable intake, family history of diabetes, hypertension, CVD, depression, and cancer.

**
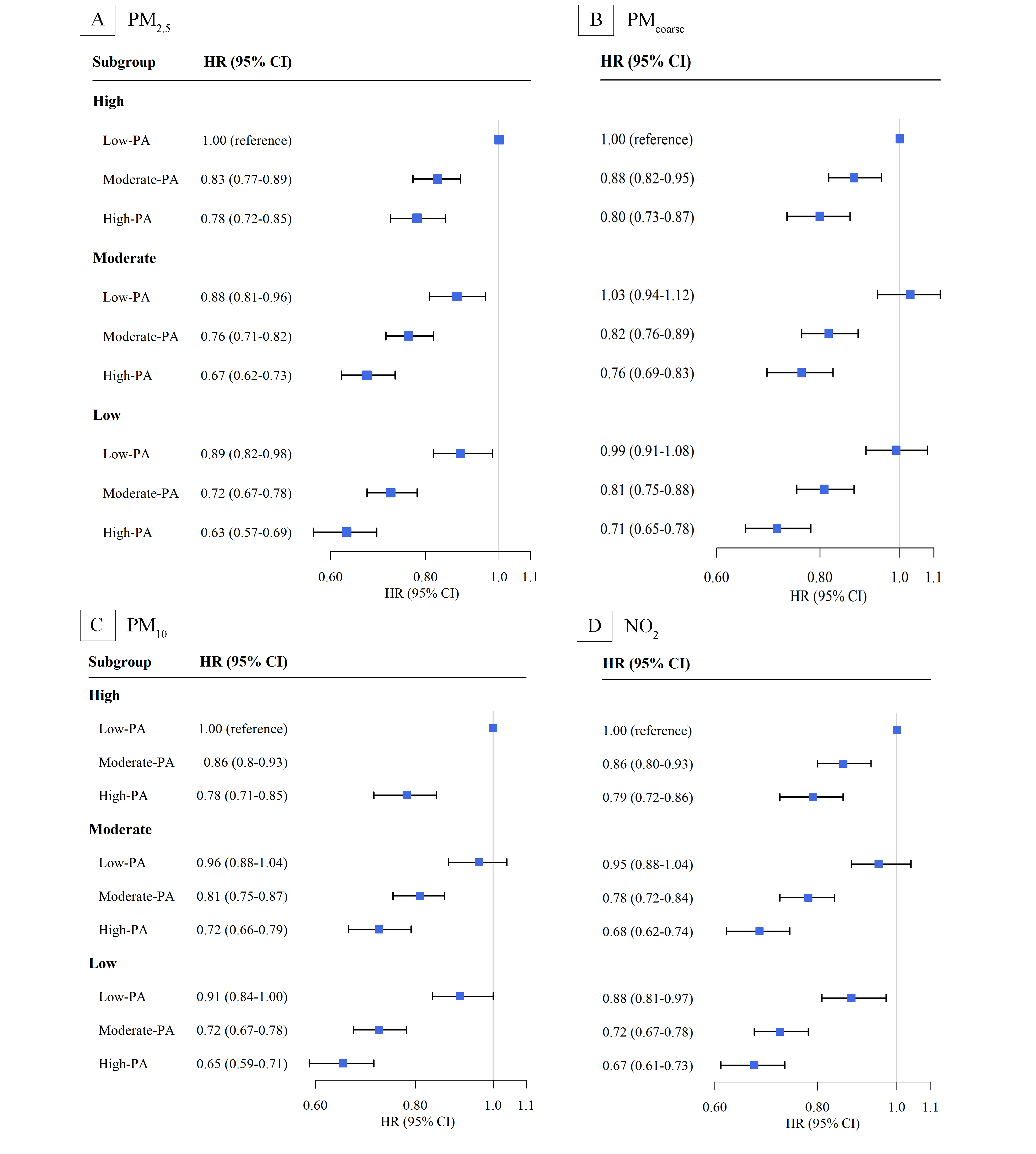
**

# Figure 4S. Joint associations of physical activity and air pollutants with incidence of type 2 diabetes after excluding participants with missing data for covariates.

Abbreviation: CI, confidence interval; HR, hazard ratio. Results obtained after adjusting age, sex race, education, household income, smoking status, alcohol consumption, BMI, and fruit and vegetable intake, family history of diabetes, hypertension, CVD, depression, and cancer.


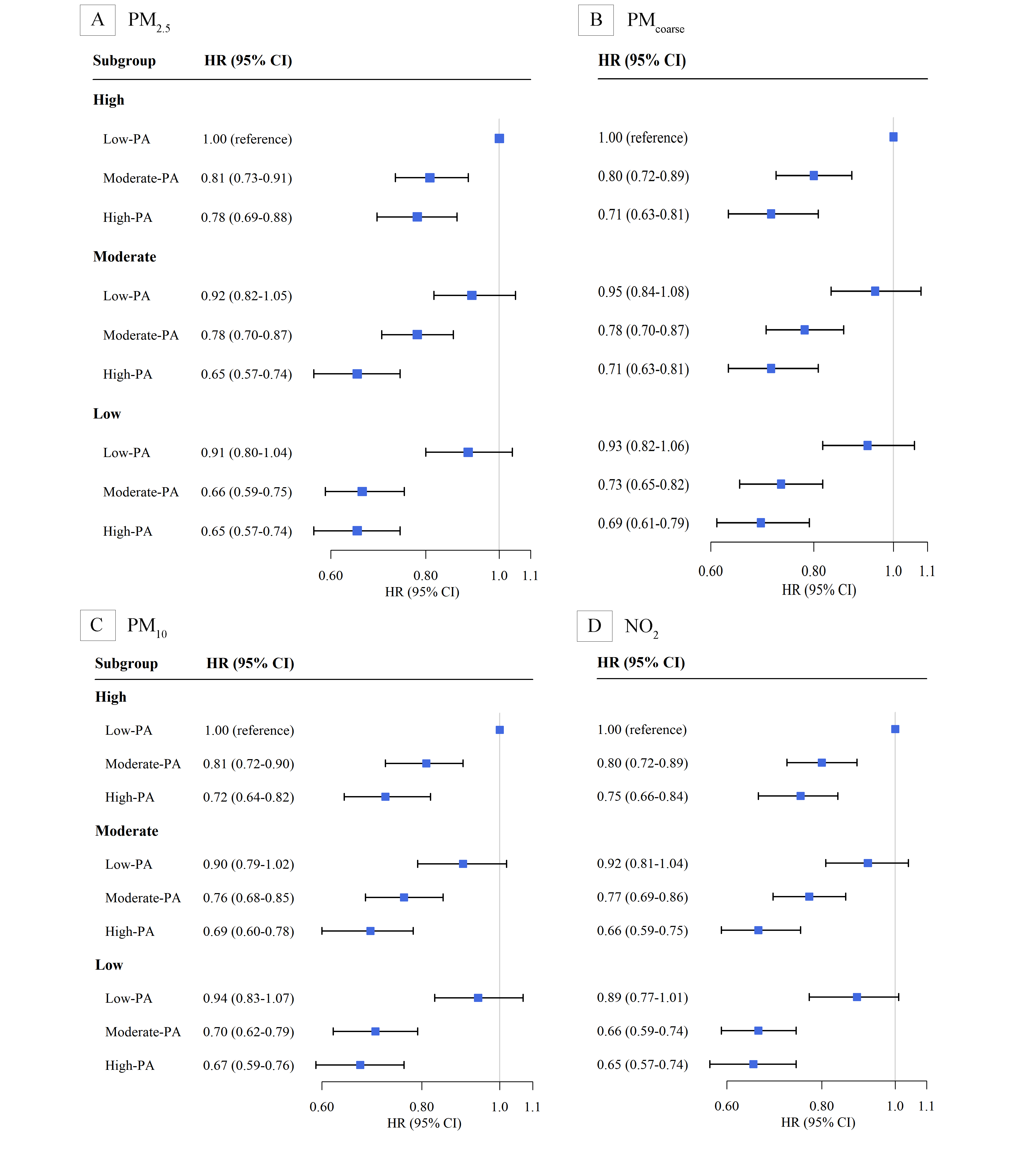


# Figure 5S. Joint associations of physical activity and air pollutants with incidence of type 2 diabetes after excluding participants who had type 2 diabetes related diseases.

Abbreviation: CI, confidence interval; HR, hazard ratio. Results obtained after adjusting age, sex race, education, household income, smoking status, alcohol consumption, BMI, and fruit and vegetable intake, family history of diabetes, hypertension, CVD, depression, and cancer.


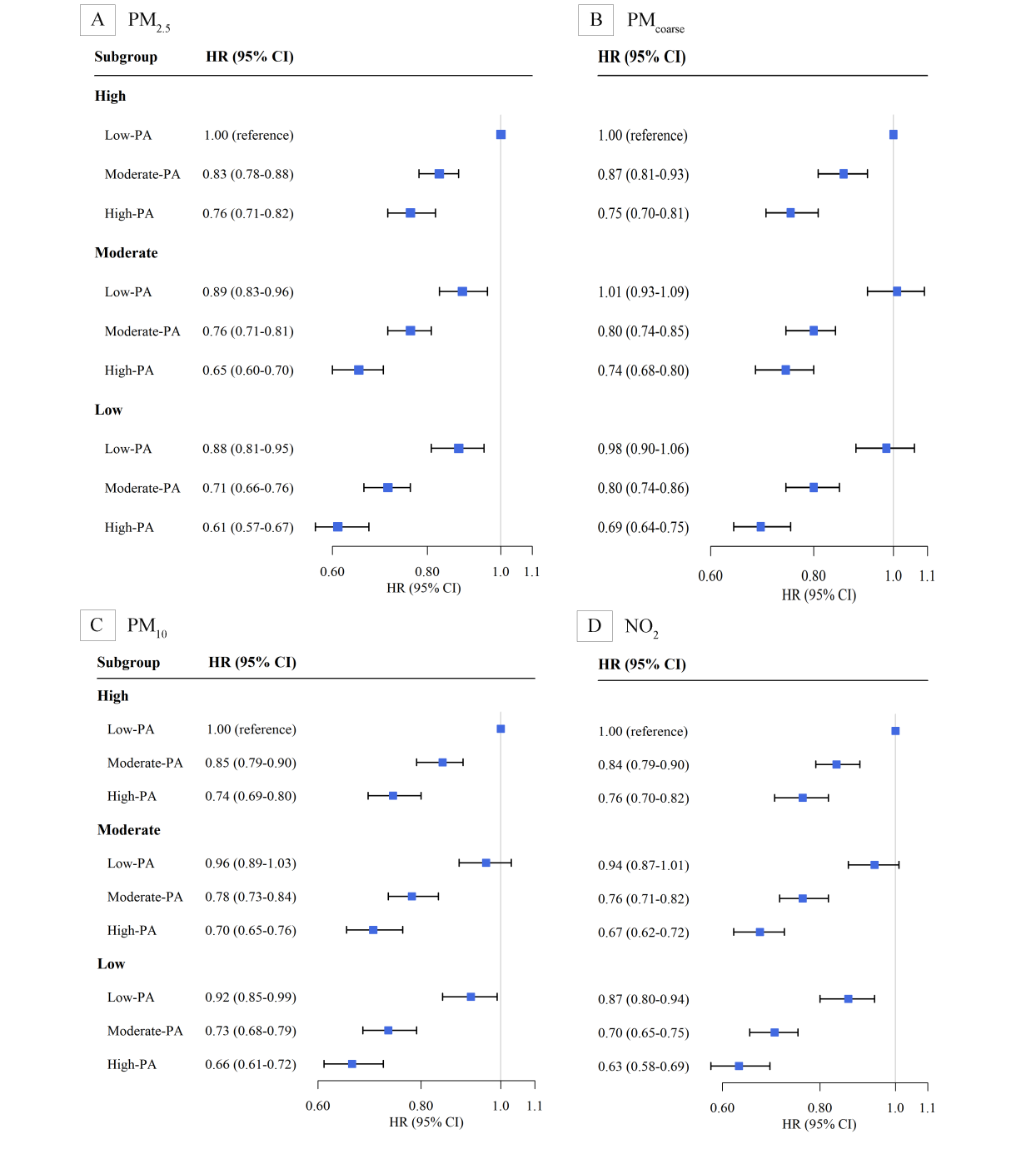


# Figure 6S. Joint associations of physical activity and air pollutants with incidence of type 2 diabetes after adjusting the employment status.

Abbreviation: CI, confidence interval; HR, hazard ratio. Results obtained after adjusting age, sex race, education, household income, smoking status, alcohol consumption, BMI, and fruit and vegetable intake, family history of diabetes, hypertension, CVD, depression, cancer, and employment status.


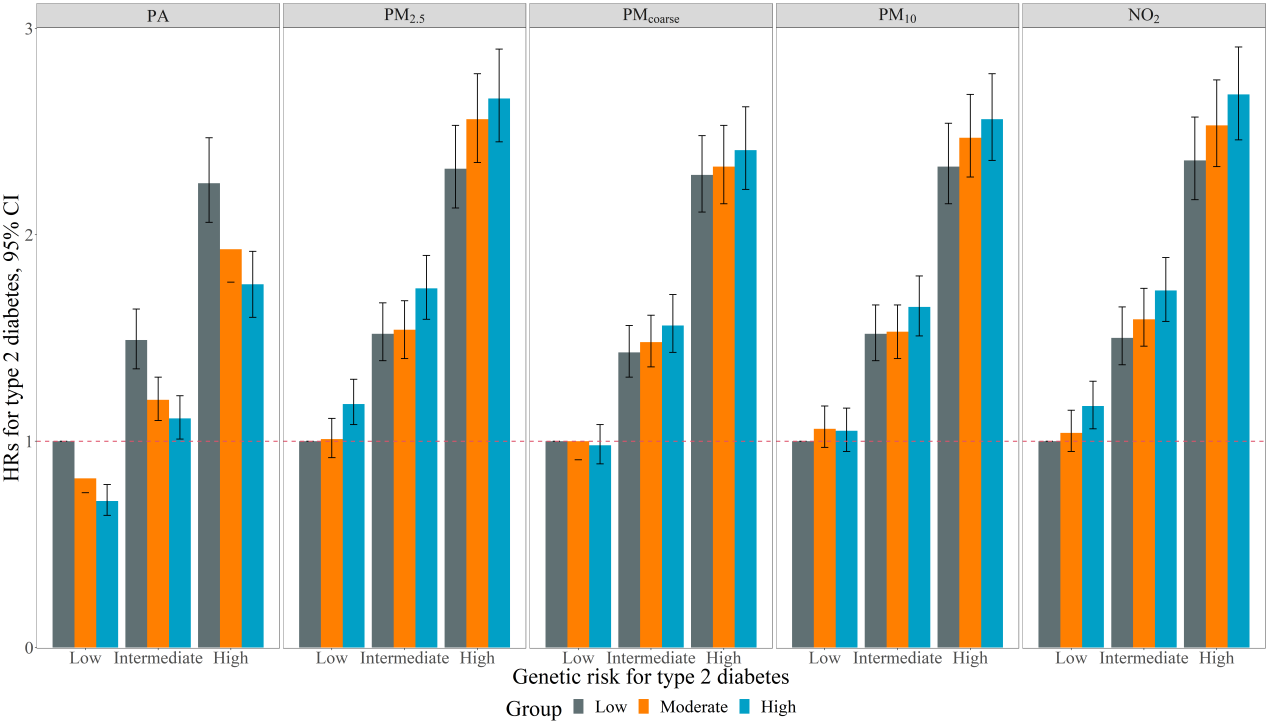


# Figure 7S. Joint associations of physical activity or air pollution and genetic risk with the incidence of type 2 diabetes after excluding participants of non-European ancestry.

Abbreviations: CI, confidence interval; HR, hazard ratio. Results obtained after adjusting for age, sex, race, education, household income, smoking status, alcohol consumption, body mass index, fruit and vegetable intake, family history of diabetes, hypertension, cardiovascular disease, depression, cancer, genotyping, the first 10 genetic principal components, and air pollution or physical activity. Individuals in the low PA or low air pollution group were used as the reference group (*).
